# When technology improves patient care and provider experience

**DOI:** 10.1016/j.rpth.2023.100088

**Published:** 2023-03-06

**Authors:** Geoffrey D. Barnes, Angela C. Weyand

**Affiliations:** 1 Frankel Cardiovascular Center, University of Michigan, Ann Arbor, Michigan, USA; 2 Mott Children’s Hospital, University of Michigan, Ann Arbor, Michigan, USA

Warfarin and other vitamin K antagonists remain commonly-used anticoagulants worldwide for both adult and pediatric patients. The need for frequent laboratory monitoring and dose adjustment present significant challenges for patients and the healthcare system. Traditional models of care rely on the health care team to review the international normalized ratio (INR) of the prothrombin time and then communicate warfarin dose adjustments to the patient. This is cumbersome for both the health system and the patients. Although technology is often billed as making everyone’s lives easier, clinicians know that the electronic health record (EHR) has often led to increased work and has been associated with higher levels of burnout [1,2].

This observational study by Jones et al. [3] quantitatively and qualitatively evaluated the use of an EHR communication tool to facilitate warfarin patient self-management (PSM) in a pediatric cohort. Participants under the age of 18 years were eligible based on prior use and adherence to home INR self-testing, a minimum of 6 months prior treatment with warfarin, and a planned duration of treatment for at least 12 months. Families attended an individualized educational session on warfarin PSM, which included review of warfarin mechanism of action, confounders to stable therapy, INR target range and dose revision, warfarin INR nomogram and rationale for dosing decisions, management of an elevated INR at home, and instructions on when to call their healthcare providers. Each session was individualized based on a preappointment knowledge questionnaire completed by the family. Participants obtained home INR values, planned their own warfarin dose adjustment using the provided nomogram (incorporating INR trend and clinical status), and entered these data in the EHR using a patient portal interface. Upper and lower limits were set for the target therapeutic range to trigger notification to the clinical team.

Of the 96 families eligible and invited to participate, 36 consented. Of those, 26 attended the training and 24 uploaded INR results to the portal during the study period and were included in the analysis. Over a 10-month period, 460 INR values were reported and the mean time in therapeutic range was 79.9% compared with 71% in the pre-PSM period, a statistically significant improvement. Among 24 families, 23 were able to adhere to the PSM program for the entire study period. Family-selected dosing agreed with the nomogram 92.6% of the time, and the recommended time for the next INR was concordant 78% of the time. No bleeding or thrombotic events occurred during the study period.

Eight families completed poststudy telephone interviews. The major theme from these qualitative interviews was empowerment, whereas minor themes of knowledge acquisition, building of confidence, efficiency, and comfort in the knowledge of a “safety net” also emerged. Empowerment is particularly important for pediatric patients near adulthood and the transition to taking primary responsibility for their healthcare. Successful transition requires adequate knowledge, a perception of self-efficacy, and self-management skills, all of which can be gained through this type of program [4]. Participation enables adolescents to engage in knowledge acquisition through real-world experience, instilling in them a sense of confidence, competency, and self-efficacy, all in the setting of a secure safety net. Further, in a world that increasingly exists “online,” the patient portal interface is likely to be well received by those in this demographic.

Similar models of care have been successfully integrated into other areas of medicine, most commonly in the field of diabetes and adult populations [5]. Digital health interventions in diabetes have been shown to be more effective in reducing HbA1C at 6 and 12 months better than usual care, although this effect is variable across groups. Although feasible and effective, a minority of eligible patients ultimately participated in the program likely because of a constellation of factors. Many patients reported satisfaction with the status quo, although this risk-benefit calculation may change over time and likely lead to increased participation.

Warfarin PSM has been tested in several studies of adults, generally showing improved warfarin control with fewer adverse events [6]. However, these generally required at least once weekly INR checks and included highly selected patient populations in the PSM cohorts.

One important aspect of PSM that has not been adequately explored is the impact that such a program may have on the workload and job satisfaction of healthcare providers. The current nursing shortage and increased turnover rate are likely to be an ongoing issue affecting all areas of healthcare. Implementation of PSM programs may shift a proportion of protocolized work to the patient, resulting in less burnout and higher job satisfaction.

The financial implications of this model of care are also largely unknown. Decreased healthcare interaction may lead to lower costs for the patient in a system that is increasingly expanding and qualifies as “billable” care but may also provide financial challenges for anticoagulation clinics financially reliant on these face-to-face interactions. Self-management models in diabetes have been shown to be cost effective, given the improvements in clinical outcomes [7]. From a clinical perspective, increased time in therapeutic range should lead to reduced bleeding and thrombotic events. Although the true rate of bleeding and thrombotic complications for pediatric patients treated with warfarin is unknown, bleeding requiring readmission has been reported in up to 2%, whereas recurrent thrombosis may occur in up to 12.9% and these events can be catastrophic [8,9]. As these programs become more commonly utilized, it will be important to continue to evaluate the effects of specific interventions. Although most reports are positive, an analysis of 6 pediatric self-management approaches for diabetes in the UK found no change in HbA1C and found that age-appropriate self-management kits inhibited self-management and increased worry in 6-18-year-old patients [10].

Although this study by Jones et al. [3] largely replicates the findings from other studies of adult patients using warfarin PSM, there are important questions about how to disseminate this care model broadly (Table). First, awareness by both patients and clinicians is a key step. Patient advocacy organizations can be very effective in this area. Second, building the technological infrastructure is critical for success. Although some of the communication features may be built into the EHRs (e.g., patient portal messaging), there remain challenges in the best way to transfer those data into structured fields for easy review by clinical teams. This opens the possibility for third-party vendors to create software that interface with the EHR and streamline communications and documentation of INR results and warfarin dosing. Third, many institutions with structured anticoagulation clinics may have policies and procedures that prevent PSM activities. As such, those policies and procedures will need to be updated. Fourth, with any technology-based intervention, addressing inequity and ensuring that healthcare disparities are not worsened is essential. Barriers include access to home INR testing equipment and reliable internet for data entry/communication. Finally, addressing financial implications, including staffing availability and anticoagulation service funding without face-to-face or phone-based visits, are important.

**Table:** Key dissemination steps

• Patient and clinician awareness of PSM • Technology infrastructure ○ Patient-EHR messaging system ○ Integrating data into existing/structured warfarin tracking systems & laboratory fields • Update anticoagulation service policies to allow PSM • Address potential issues of inequity ○ Access to home testing meters ○ Access to reliable internet/communication tools • Identify funding model for staff that does not rely on face-to-face or phone-based visits

EHR, electronic health record; PSM, patient self-management

We applaud the authors for their innovative work that empowers patients and their families, reduces the work burden on the healthcare system, and demonstrates improved clinical management. We are especially excited to see this work conducted in the pediatric population where additional efforts are needed to understand and improve care delivery models. Furthermore, we believe that addressing technology barriers and issues of healthcare inequity will be essential for broad dissemination.

## References

[1] Hilliard R.W., Haskell J., Gardner R.L. (2020). Are specific elements of electronic health record use associated with clinician burnout more than others?. J Am Med Inf Assoc.

[2] Kroth P.J., Morioka-Douglas N., Veres S., Babbott S., Poplau S., Qeadan F. (2019). Association of electronic health record design and use factors with clinician stress and burnout. JAMA Netw Open.

[3] Jones S., Hislop J.L., Gilmore H., Greenway A., Hibbard J.H., Monagle P., Newall F. (2023). Using an electronic medical record patient portal for warfarin self-management: Empowering children and parents. Res Pract Thromb Haemost.

[4] John A.S., Jackson J.L., Moons P., Uzark K., Mackie A.S., Timmins S. (2022). American Heart Association Adults With congenital Heart Disease Committee of the Council on Lifelong congenital Heart Disease and Heart Health in the Young and the Council on Clinical Cardiology; Council on Cardiovascular and Stroke Nursing; Council on Arteriosclerosis, Thrombosis and Vascular Biology; and Stroke Council. Advances in Managing Transition to Adulthood for Adolescents With Congenital Heart Disease: A Practical Approach to Transition Program Design: A Scientific Statement From the American Heart Association. J Am Heart Assoc.

[5] Chatterjee S., Davies M.J., Heller S., Speight J., Snoek F.J., Khunti K. (2018). Diabetes structured self-management education programmes: a narrative review and current innovations. Lancet Diabetes Endocrinol.

[6] Dhippayom T., Boonpattharatthiti K., Thammathuros T., Dilokthornsakul P., Sakunrag I., Devine B. (2022). Clinical outcomes of different warfarin self-care strategies: a systematic review and network meta-analysis. Thromb Haemost.

[7] Ye W., Kuo S., Kieffer E.C., Piatt G., Sinco B., Palmisano G., Spencer M.S., Herman W.H. (2021). Cost-effectiveness of a diabetes self-management education and support intervention led by community health workers and peer leaders: projections from the racial and ethnic approaches to community health detroit trial. Diabetes Care.

[8] Andrew M., David M., Adams M., Ali K., Anderson R., Barnard D., Bernstein M., Brisson L., Cairney B., DeSai D. (1994). Venous thromboembolic complications (VTE) in children: first analyses of the Canadian Registry of VTE. Blood.

[9] Moffett BS, Kim S, Bomgaars LR. Readmissions for warfarin-related bleeding in pediatric patients after hospital discharge. *Pediatr Blood Cancer*. 2013;60:1503–6. 10.1002/pbc.24546 23606286

[10] Noyes J., Allen D., Carter C., Edwards D., Edwards R.T., Russell D., Russell I.T., Spencer L.H., Sylvestre Y., Whitaker R., Yeo S.T., Gregory J.W. (2020). Standardised self-management kits for children with type 1 diabetes: pragmatic randomised trial of effectiveness and cost-effectiveness. BMJ Open.

